# ERRATUM: Effects of dipeptidyl peptidase-4 inhibitor in insulin-resistant rats with myocardial infarction

**DOI:** 10.1530/JOE-16-0096e

**Published:** 2025-11-10

**Authors:** Nattayaporn Apaijai, Tharnwimol Inthachai, Suree Lekawanvijit, Siriporn C Chattipakorn, Nipon Chattipakorn

**Affiliations:** ^1^ Cardiac Electrophysiology Research and Training Center, Faculty of Medicine, Chiang Mai University, Chiang Mai, Thailand; ^2^ Cardiac Electrophysiology Unit, Department of Physiology, Faculty of Medicine, Chiang Mai University, Chiang Mai, Thailand; ^3^ Center of Excellence in Cardiac Electrophysiology Research, Chiang Mai University, Chiang Mai, Thailand; ^4^ Department of Pathology, Faculty of Medicine, Chiang Mai University, Chiang Mai, Thailand; ^5^ Department of Oral Biology and Diagnostic Sciences, Faculty of Dentistry, Chiang Mai University, Chiang Mai, Thailand

The authors and journal apologise for an error in the above paper, which appeared in volume 229, part 3, pages 245–258 . The error relates to Fig. 5, given on page 255, in which the blots shown in panel D were inadvertently labelled incorrectly. In panel D, the blot labelled ‘p-TGF-β’ should have been labelled as ‘TGF-β’ and the blot labelled ‘TGF-β’ should have been labelled as ‘Actin’. This error had no impact on the results, interpretation or conclusions of the paper.

The corrected Fig. 5 is given in full below:

**Figure 5:**
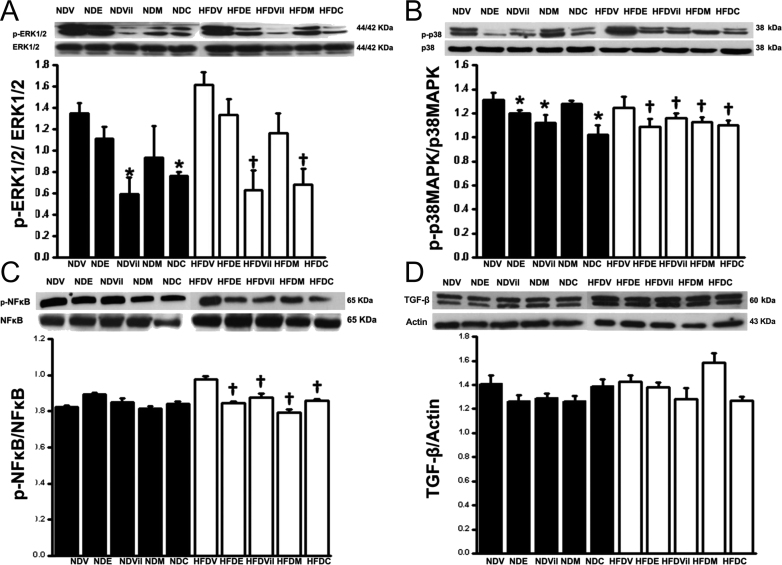
Effects of enalapril, vildagliptin, metformin, and combined drugs on p38 MAPK phosphorylation, ERK1/2 phosphorylation, NF-κB phosphorylation, and TGF-β expression in normal and obese-insulin-resistant rats after chronic MI. Vildagliptin and combined vildagliptin and metformin reduced ERK phosphorylation in ND and obese-insulin-resistant rats (A). Enalapril, vildagliptin, metformin, and combined vildagliptin and metformin reduced p38 MAPK phosphorylation in obese-insulin-resistant rats. In ND rats, enalapril, vildagliptin, and combined vildagliptin and metformin but not metformin reduced p38 MAPK phosphorylation (B). Enalapril, vildagliptin, metformin, and combined vildagliptin and metformin reduced NF-κB phosphorylation in obese-insulin-resistant rats (C). TGF-β expression was not different among groups (D), *P < 0.05 vs NDV, ^†^*P* < 0.05 vs HFDV. NDV, normal diet rats treated with vehicle; NDE, normal diet rats treated with enalapril; NDVil, normal diet rats treated with vildagliptin; NDM, normal diet rats treated with metformin; NDC, normal diet rats treated with combined drugs; HFDV, high-fat-fed rats treated with vehicle; HFDE, high-fat-fed rats treated with enalapril; HFDVil, high-fat-fed rats treated with vildagliptin; HFDM, high-fat-fed rats treated with metformin; and HFDC, high-fat-fed rats treated with combined drugs.

